# Women’s perceptions of effects of war on intimate partner violence and gender roles in two post-conflict West African Countries: consequences and unexpected opportunities

**DOI:** 10.1186/1752-1505-8-12

**Published:** 2014-08-04

**Authors:** Rebecca Horn, Eve S Puffer, Elisabeth Roesch, Heidi Lehmann

**Affiliations:** 1 Institute of International Health and Development, Queen Margaret University, Edinburgh E21 6UU, UK; 2 Department of Psychology and Neuroscience, Duke University, Box 90086, 417 Chapel Drive, Durham, NC 27708-0086, USA; 3 International Rescue Committee, 22 East 42nd Street, New York, NY 10168, USA

**Keywords:** West Africa, Intimate partner violence, Domestic violence, Post-conflict

## Abstract

**Background:**

The aim of this paper is to explore women’s perceptions of the causes of intimate partner violence (IPV) in West Africa, and the ways in which they understand these causes to interact with the experiences of war.

**Methods:**

The study was conducted in two locations in Sierra Leone and two in Liberia, using focus group discussions (N groups =14) and individual interviews (N = 20).

**Results:**

Women perceive the causes of IPV to be linked with other difficulties faced by women in these settings, including their financial dependence on men, traditional gender expectations and social changes that took place during and after the wars in those countries. According to respondents, the wars increased the use of violence by some men, as violence became for them a normal way of responding to frustrations and challenges. However, the war also resulted in women becoming economically active, which was said by some to have decreased IPV, as the pressure on men to provide for their families reduced. Economic independence, together with services provided by NGOs, also gave women the option of leaving a violent relationship.

**Conclusions:**

IPV was found to be a significant problem for women in Sierra Leone and Liberia.

The interactions between war experiences and financial and cultural issues are multi-faceted and not uniformly positive or negative.

## Background

West Africa is a region that is slowly emerging from multiple, interconnected wars in which violence against women and girls was prevalent [1,2]. While sexual violence both during and in the wake of these wars has received much attention, less priority has been given to intimate partner violence (IPV) in relation to war^a^. Although empirical research in this field is limited, studies have suggested a link between exposure to violence by armed groups and IPV [3,4]. The aim of this paper is to explore women’s perceptions of the causes of IPV in West Africa and the ways in which they believe these causes interact with the experience of war.

### The influence of cultural and social norms on IPV

Certain cultural and social norms play a particularly important role in creating conditions that support or facilitate IPV [5]. These include the belief that a man has the right to assert power over a woman and is socially superior; that a woman’s freedom should be restricted; that physical violence is an acceptable way to resolve conflict within a relationship; and that divorce brings shame to the family [5].

Many of these norms are common in parts of sub-Saharan Africa, resulting in IPV being widely justified by both men and women as a normal part of an intimate relationship [6,7]. In many African settings, the majority of both men and women have been found to agree that a husband has the right to use violence in response to women’s transgression of traditional gender roles, such as if she is disobedient, fails to perform household and child care duties, or is unfaithful [8-10]. Indeed, a study in rural Kenya found that while women did seek, and sometimes receive, support for IPV from informal community resources, the prevailing opinion was that this violence within partnerships was a generally accepted aspect of local culture [11]. A study analysing the Ugandan DHS data found that the majority of women (73%) and men (57%) believed that wife beating is acceptable in at least one of the following scenarios: if the wife goes out without informing her husband; if she neglects the children; if she argues with the husband; if she refuses to have sex with the husband; or if she burns the food [12]. In one specific community in Sierra Leone, the belief that a woman’s prayer goes to God through a man creates a situation in which every woman must be affiliated to a man if she is to find acceptance in the community [13]. While this particular belief is not common across all communities, it is illustrative of the complex interactions between gender roles, spirituality, and social norms that can affect women's well-being.

The transgression of gender norms and failure to fulfil cultural expectations of good womanhood and successful manhood are among the most important triggers for IPV [2]. For example, Mann & Takyi [14] write that frequently-cited reasons for marital violence relate to women behaving in a way unexpected of their gender, such as neglecting household chores, disobeying husbands or elders, and withholding sex from partners. Many have suggested that violence occurs when men are unable to live up to the socially presented ideas of what it means to be a ‘successful’ man [15,16]. Olayanju et al. [6] note that certain aspects of life in sub-Saharan Africa make IPV more likely, including widespread poverty that makes it difficult for men to achieve what is expected of them socially.

In addition, there is some evidence that the process of cultural change, especially shifts in gender roles and expectations, is related to increased IPV. The WHO [17] cited the ‘sanctions and sanctuary’ framework, which suggests that IPV may be highest in societies where the status of women is in a state of transition: where women have a very low status, violence is not needed to enforce male authority, and where women have a high status, they will probably have achieved sufficient power collectively to change traditional gender roles. It is the middle ground where violence can spike. Studies of the short-term effects of micro-credit programmes aimed at empowering women may provide helpful examples of these transition periods. They have found mixed results in terms of the impact on IPV [18], with some finding that economic empowerment protects women from IPV whilst others report that women’s adoption of non-traditional roles creates tension in the home and precipitates IPV.

### The influence of conflict on IPV

Whilst the relationship between IPV and conflict remains under-studied, there is evidence that IPV has increased during periods of conflict in Afghanistan, Lebanon, Palestinian territories, Sri Lanka, and Côte d’Ivoire [19-22]. In Côte d’Ivoire, Hossain and colleagues documented that IPV was the most frequently reported form of violence following the period of armed conflict (20.9% among women; 9.9% among men) [22]. Vinck and Pham [23] detail the evidence in support of a hypothesis that there is a relationship between conflict and IPV, including studies that have found higher rates of IPV amongst war veterans compared to civilian couples, and they trace the relationship between war experiences, subsequent mental health problems and increased likelihood of either perpetrating or experiencing IPV.

There are a number of pathways through which war experiences could potentially contribute to IPV. Annan & Brier [24] note the role played by structural factors that permit and sustain IPV in war-affected areas. In conflict settings, kinship support systems may become fractured as people are displaced; judicial systems and institutionalised conflict-resolution mechanisms no longer function effectively, and a culture of impunity may develop [25]. Although these factors are not unique to such settings, they are undoubtedly exacerbated by prolonged conflict.

The experience of conflict affects almost every aspect of socio-economic life. Poverty is amplified, and there are often limited opportunities to generate income; these stresses have the potential to contribute to IPV [26]. In a study of refugees from nine different countries, poverty was cited repeatedly as a cause of violence [27]. A man’s inability to fulfil the traditional ‘provider’ role was said to contribute to feelings of anger and shame, which manifest themselves in violence, especially when his wife or children ask for things he is unable to provide.

A loss of social role and status is a common consequence of war and displacement, as people lose their jobs, access to land for farming, and their roles within their communities [28-30]. This is particularly true for men. The nature of gendered division of labour in most societies is that men’s roles and masculinity are tied to participation within the public realm, while women’s roles and femininity are linked to the personal or household sphere. Women are often able to maintain their traditional roles and responsibilities, and so maintain their gender identity, whilst men are more likely to lose their central roles, especially that of protector and provider, which makes it difficult to maintain their sense of themselves as men in the community and family [28,31]. Some have found connections between these losses and a tendency towards marital conflict, especially where women take on the role of breadwinner [25-27,32,33].

A society that lives with conflict and war may be more likely to develop social norms in which violence is seen as a normal way to resolve problems, and, therefore, in which IPV is more likely. Uthman et al. [7], in their study of 17 sub-Saharan African countries, found that women were more likely to experience IPV in societies where the use of violence is a socially accepted norm. The WHO [17] states that the normative use of violence to resolve conflicts is one of two specific risk factors that have been found to be strongly associated with IPV (the second is the unequal position of women in a particular relationship and in society).

One of the few studies of the relationship between experience of war and IPV (focusing only on physical violence) was conducted by Vinck & Pham [23] in Liberia. Their national multi-stage cluster survey of 4501 adults (2196 of whom were female) found exposure to war-related violence to be associated with increased likelihood of intimate-partner physical violence. Men who had direct experience of war-related potentially traumatic events, or who had witnessed or taken part in the conflict, were more likely to perpetrate IPV. Vinck and Pham suggest that this may reflect the impact that violence has on individuals’ socio-economic characteristics and on their belief and value systems. The qualitative study reported in this paper builds on the work of Vinck and Pham, amongst others, by exploring in some depth the relationship between conflict and IPV from the perspective of women who experienced the wars in Liberia and Sierra Leone and were living in post-conflict communities.

### Conflict in Sierra Leone and Liberia

Sierra Leone is located on the west coast of Africa, bound on the north by Guinea, the south-east by Liberia and the south-west by the Atlantic Ocean. The population in 2012 was estimated to be close to 5.5 million [34].

Sierra Leone has experienced political instability since the late 1960s, but protracted armed conflict began in 1991 when the Revolutionary United Front tried to topple the constitutionally elected government by launching a guerrilla war from neighbouring Liberia. During the conflict, tens of thousands died and more than 2 million people were displaced, either to another part of Sierra Leone, into a neighbouring country or further afield. The war was characterised by mutilations and forced recruitment of children, as well as killings, sexual assault, looting and destruction of property. The Lome Peace Accord was signed in July 1999, the first dispatch of United Nations soldiers arrived soon after, and disarmament of rebel forces began the following year. In January 2002, President Kabbah declared that the civil war was officially over.

There has been no recent study of the prevalence of IPV in Sierra Leone, but in 1998 66.7% of 144 women surveyed reported having been beaten by an intimate partner [35]. Sierra Leone passed a domestic violence act in 2007, establishing basic rights for women in the home and entitlements for survivors such as free medical care, but implementation of the act has been limited [36].

Liberia is on the coast of West Africa, bordering Guinea, Ivory Coast and Sierra Leone. It has a population of approximately 4 million (estimated in 2009) [37]. The country experienced a long and divisive civil war, beginning in 1989 and ending with the signing of a peace treaty in 2003. It is estimated that more than 250,000 people were killed in fighting or massacres and one million people (one third of the population) were displaced [1]. Towns and cities were devastated, and most of the country’s infrastructure was destroyed.

Studies among the Liberian population have found high levels of IPV [38]. A survey of 4526 mothers found an incidence rate of 40% for emotional and physical IPV combined [39], and prevalence rates of 31.5-50.1% were found for physical IPV amongst sub-groups of Liberian women [40]. A national multi-stage cluster survey found that 37.7% of women reported lifetime exposure to intimate partner physical violence and 24.4% reported incidence of intimate-partner physical violence over a one-year recall period [23].

In this paper we report on women’s perceptions of the causes of IPV in West Africa and the ways in which they understand these causes to interact with the experiences of war.

## Methods

The research team visited four locations in July 2011: Freetown and Kailahun in Sierra Leone, and Voinjama and Monrovia in Liberia. Two methods of data collection were used: focus group discussions and individual interviews. This study was conducted in collaboration with the International Rescue Committee (IRC)^b^, a humanitarian organization that made arrangements for data collection as part of an advocacy effort to inform programming and policies related to IPV in West Africa [41].

### Participants

IRC staff in each location recruited participants from the women who were receiving support services from the IRC. The criteria used to select participants for focus group discussions were that participants should be women over 18 and living in the target community, and that there should be diversity in terms of age and socio-economic status. They were not selected according to whether or not they had experienced IPV. Participants in Freetown and Monrovia lived in an urban setting, and those from Voinjama and Kailahun were from a rural setting.

### Focus group discussions

The purpose of the focus group discussions was to explore the nature of IPV in that community, beliefs about causes and effects, options available to women experiencing IPV, and women’s help-seeking behaviour. Women were asked to share their observations of the situation in their community, rather than their own personal experiences, though in many cases women chose to share their own experiences of violence.

The term ‘domestic violence’ was commonly used in both Sierra Leone and Liberia, so was used during interviews and focus group discussions. However, we specified that we were asking about violence from an intimate partner (husband or boyfriend), rather than from other family members.

The facilitator followed a semi-structured focus group guide, with a small number of questions used to structure the discussion. These questions were not asked directly, but were explored through a broader discussion of relevant issues, including through scenarios and examples, often presented by the participants themselves.

Fourteen focus group discussions were conducted: five in Freetown, four in Kailahun, three in Monrovia and two in Voinjama. All groups except two consisted of 8 women; the total number of participants was 110. Some participants were members of IRC-facilitated Women’s Action Groups. We interviewed two groups of professional women (one in Freetown and one in Kailahun) and one group of women working in the local market (in Kailahun); the others were general members of the community who were not grouped according to any specific criteria. Most discussions took place in a Women’s Centre, with some being conducted in the IRC office and one in a school classroom. The length of the interviews ranged from 66 to 88 minutes (mean = 78 minutes).

### Individual interviews

Women survivors of IPV were interviewed to explore individual experiences of IPV and their opinions on the causes of specific perpetrators’ behaviour. Interviews began with a broad question which enabled women to recount their experiences in their own way (e.g. ‘tell me the story of the violence you experienced and the impact it has had on your life’). Depending on the response to this opening question, the interviewer probed to explore the issues of interest, which included the woman’s perception of the triggers for her partner’s violence. The interview was semi-structured, with the interviewer exploring certain themes in a fluid and dynamic way and following up on any other issues of interest raised by the interviewee.

Twenty individual interviews were conducted: five in Freetown; five in Kailahun; four in Voinjama; and six in Monrovia. Women living in both urban and rural settings were interviewed, and their ages ranged from 18 to 49 (mean age = 30 years). Some were married and had experienced violence over many years; some were single and had been beaten by their boyfriends regularly; and three were single and had experienced a single very severe assault at the hands of their boyfriends.

The interviews were conducted in a location chosen by the woman. Sometimes this was the IRC office; in other cases it was a friend’s house, the IRC vehicle, a Women’s Centre, or the woman’s workplace. The length of the interview ranged between 17 and 68 minutes (mean = 38 minutes).

### Translation and transcription

Our procedure for translation followed the example of Pavlish [42] in which the lead researcher asked questions in English and a translator repeated the question in the local language. The participant responded in their local language, and the translator immediately translated that response into English. The speech of the translator was recorded, and the lead researcher took her own notes in English.

The recordings were transcribed after the interview by the lead researcher and then deleted from the voice recorder and the computer on which they were played. All participants were asked whether they were comfortable with the discussion or interview being recorded, and none expressed any concern.

### Analysis

Analysis was conducted by the lead researcher in two stages. Whilst in the field, a rapid analysis was conducted, in which the researcher identified key themes in the data based on the research questions. She then reviewed the data under each of these themes and identified the key issues within each theme. The issues were summarised to present during debrief meetings which took place in each location. IRC staff, research participants and other community members took part in the debrief meetings, during which the initial findings were discussed and explored. This process enabled the lead researcher to check her initial analysis, learn from the discussion of the initial findings, and ensure that it reflected participants’ understanding of the key issues.

Once the data collection stage was over, analysis was conducted using complete data from all locations, both the fully transcribed interview data and feedback obtained during the debrief meetings. The thematic analysis undertaken for the initial analysis was used to define a set of conceptual codes that was then systematically applied to all text using Nvivo9. New codes were added as they emerged, and the initial list of codes was revised until it accurately reflected the data set. The data within each code were then comprehensively reviewed, and patterns between codes explored.

### Ethical issues

There were significant ethical considerations involved, and the safety and wellbeing of women was prioritised. The research was planned and conducted in accordance with the WHO’s guidelines [43] on conducting research on domestic violence. A set of safety guidelines were developed, which consisted of the following elements:

• Informed consent sheets were used to explain verbally to participants what they needed to know before they decided whether or not to participate. The individual interviewees signed these forms, or gave their thumbprint, if they consented to participate after being given all the relevant information, and having an opportunity to ask questions. In the case of the focus group discussions, the lead researcher signed each form to confirm that the information had been given to the women and that they had agreed to participate.

• In all locations systems were in place to provide additional support to participants if required. Women participating in individual interviews were already in contact with IRC so were able to access services if required. Contact details of relevant services were provided to women participating in focus group discussions.

• An ethical check was conducted, which involved IRC staff checking on participants a few days after the interview to find out whether ‘anything good or bad has happened as a result of talking to us’. This check was repeated after two weeks and one month.

• Systems to ensure that a woman’s information was kept confidential (e.g. no names were recorded) and that she was not identified as a survivor by being invited to participate in the study.

• All participants were told how to contact someone they could talk to if they had further questions about the study or wish to withdraw.

The secondary analysis of these data and publication of these findings was approved by the Institutional Review Board at Queen Margaret University, UK.

## Results

Women described the factors that contributed to IPV in post-conflict Sierra Leone and Liberia as being inter-related in complex ways and commonly described IPV as part of a cluster of behaviours, including the man’s infidelity and failure to provide for his family. The majority of women’s responses were focused on cultural and financial issues as two overarching categories, which underlie many of the causal factors they identified. We first describe these overarching issues and then the specific ways in which experiences of war interact with them to influence IPV.

### Cultural models and social norms

Underlying much of the discussion around factors that contribute to IPV in Sierra Leone and Liberia were issues relating to traditional culture, particularly as it relates to gender roles and expectations. Respondents related two traditional cultural beliefs in particular to IPV: firstly the belief that a woman is her husband’s property, especially if he has paid dowry; and secondly the belief that the man has total authority in the home and his decisions and actions are not to be questioned by his partner.

Even if a man he came late, and a woman asks him ‘where are you coming from?’, he will beat her for that. ‘Why should you ask me? I’m the one who should control you, you don’t have the right to ask me’. So you get beaten for that. ‘I’m the head of the household. I’m the head of this home, you don’t ask me any questions. Your duty is to sit in this home’. (Voinjama group 1)

Whilst our findings suggest that many men and women in Sierra Leone do not adhere strictly to these traditional beliefs, the respondents said that when men did hold these beliefs strongly, IPV was more likely.

#### Women questioning men’s authority: a trigger point for violence

Given the cultural belief that males have absolute authority in the home, it is risky for women to give any negative response to a man’s behaviours or decisions. Male infidelity is common in both Sierra Leone and Liberia, and IPV was often said to occur when a woman challenged her partner over his extra-marital relationships, or over the money he spends on girlfriends. In some cases, women’s husbands told them that they had taken a second wife, and when the woman refused to accept the second wife into her home, she was beaten.

Other challenges said to commonly trigger violence included a woman asking her partner for money or questioning his use of money; or complaining about his staying out late; or drinking excessively. A man’s angry response to such questioning is linked to gender norms which construct men as having the right to do what they choose without giving an explanation to their partner.

He doesn’t sleep in the house and when he comes in the morning and people try to tell him, he will insult them, and when I talk he begins to beat me. Sometimes he takes stick and beats me. Whenever he sleeps out and I ask him he beats me – whenever I ask him about anything, he beats me. If there’s no soap to wash the baby things, no food money, if I ask him he will beat me. (Monrovia 19)

A woman’s attempt to participate in decision-making in relation to household resources was also said to be a trigger for violence in some relationships.

Men don’t understand about women’s rights in the house, like decision-making. If you have cultivated groundnuts, and you have harvested it, the one to sell it needs to engage me in decision-making. If he sells that thing now, and I come and ask for it, and he tells me ‘you are not controlling the house, I’m the head of the house’. Then you quarrel because you’re not seeing the money and both of us cultivated it; it was a joint something, and now you’re saying you’re the head of the house, we quarrel. The end result is beating. The men always think that they have rights. (Kailahun group 2)

In addition to verbal questioning of her partner’s behaviour, a woman was also said to risk a violent response if she questioned her partner’s authority by refusing to have sex or to carry out tasks.

Some women, when they’re aware that their husbands have girlfriends, they want to protect themselves. And if they do, that’s where the fight comes; if they refuse their man sex he’ll beat her. (Freetown group 2)

### Household economic relations

Financial issues were described to be a central factor in many violent relationships. IPV often occurred when a man was not supporting his family, either because he was unable to or because he spent his money on activities outside the home (typically girlfriends and/or drinking alcohol). In some situations, women attributed a man’s anger to his partner questioning his use of household resources and money. In others, they described that a man’s anger is sometimes due to shame that he was unable to support his family in the way that a man should. However, some respondents pointed out that not all men who were struggling financially responded violently when asked for money at home; some were able to explain the constraints they were under to their wives, and the couple would find a solution together. Thus, women did not see poverty as the direct cause of IPV; rather, they observed interactions between poverty and men’s beliefs about gender roles and their ability to communicate with their partners.

Sometimes you see the husbands don’t have financial means to support the home, and the wife is trying to talk of the children’s school fees, trying to talk of the feeding of the home, and you will see men getting so angry and chopping on the wife and fighting. Because of financial support. Because he don’t have to give, and then he sees the woman asking him to give, so he’ll just get angry because it’s shaming him. He feels ashamed and jumps on the wife, ‘you’re supposed to be down, you’re not supposed to give me hard time’, he will jump on the wife and start to beat her … [But] some, if he will not have, you ask them for support or for tuition, they will take their time and explain, not all of them are violent. (Monrovia group 2)

#### Women’s financial dependence on men

According to our respondents, women’s financial dependence on men contributes to IPV in a number of ways. Firstly, if a woman has no income of her own, she is forced to ask her partner for money to meet all of her needs and those of her children, which was said to be a common trigger for violence. A woman who has no independent source of income has fewer options if her relationship becomes violent; it is difficult for her to leave regardless of how badly she is treated. This may lead her partner to use violence with no fear of any repercussions; the man knows that she will not leave, report him to the police, or take any other action that may jeopardise the relationship.

When you are dependent on the man completely, you can’t leave. He can do anything to you. You have to remain there because you are dependent on him. That’s the reason some women can remain there until they get killed. (Voinjama group 2)

A group of women who are particularly vulnerable to IPV are young women from poor families. When the girl’s family is unable to provide for her, she may feel forced into relationships with men who are in a position to provide; her complete dependence on the man leaves her vulnerable to abuse.

### Direct effects of war on IPV

Women were specifically asked about how the wars in Sierra Leone and Liberia had affected IPV. Very few of the survivors interviewed individually believed that their own abusive partner’s behaviour was influenced by the war.

He didn’t join the rebels or the government troops. He’s just a wicked man. It [the war] had no effect at all. (Kailahun 8)

Some participants in group discussions also felt that the experience of war had not affected men’s use of violence towards women because they had seen the same behaviours even before the war. However, the majority in the group discussions reported that the war did influence IPV in several ways. Women described direct effects of war on IPV, as well as how social changes related to the war affected IPV.

A number of the women we spoke to felt that many of the men who had fought in the war, or who had accompanied the fighters, had been permanently affected by their experiences. They had come to see violence as a normal way of responding to challenges or frustrations and as an appropriate way of getting what they wanted. Former fighters who had been used to commanding others and getting material goods by force were said to be frustrated because they were no longer able to fulfil their needs so easily and to take out this frustration on their wives or girlfriends.

Some men are used to the weapons, they are still used to the violent behaviour, they are used to getting things free of charge, they don’t pay for it. And as a result, when things are normal now, they are not getting those free things, and you are staying with them, it will just result in violent behaviour. They were very used to looting of property, you know, and now they don’t have anything. So if you’re in a relationship with those kinds of men, you the woman, you are in trouble. Maybe he wants a small thing, maybe he wants to smoke a cigarette, he does not have money, it results into anger and then the only thing he has to do is use his fists. (Kailahun group 1)

In addition, those who had committed or seen sexual violence during the war were thought to have reduced respect for women and thus be more likely to mistreat them. Those who were children at the time of the war and were involved either as combatants or as followers were said to have been particularly affected by their experiences.

The child soldiers. The rebels, their followers that didn’t hold the gun, they are no longer boys. And now that they learned all that violence, they have grown up now. Most of these boys, they were the followers, they were carrying the loads of the fighters, so they were seeing how they were brutalising people, beating them to remove their property, to rape women. They have seen all those things during the war, and they were young boys. Now that they have grown up, they are doing it, and they are doing it more than those who were doing it during the war. (Kailahun group 3)

Some of those who used drugs during the war were said to have ongoing psychological effects, and some continued to use drugs even after the war, which our respondents believed increased their violent behaviour.

During the war some men took drugs, like cocaine, marijuana, and they are still in that habit. So after the war, maybe you go into a small argument, and the end result is fighting. Most of them fought during the war, in fact we have so many rebels here, and most of them were taking drugs. Every day they fight. If they don’t quarrel with their wives in the morning, they quarrel in the afternoon or in the evening. (Kailahun group 2)

Some of those who did not fight in the war were also said to be directly affected in ways that increased the likelihood of their perpetrating IPV. The distressing experiences that occurred during the war, particularly the loss of loved ones and of property, were said to make it difficult for some people to tolerate additional stresses. Women said that men who experienced these reactions to the consequences of war were sometimes more likely to respond to challenges in the home with violence.

With a conflict you find that so many people are distressed. And if a man is distressed, and a woman comes bothering him with regards to food for the home and that kind of thing, or asking for other kinds of support, definitely the man, rather than trying to get the woman to see reason as to his situation, the reaction is either verbal violence or physical violence. (Freetown group 1)

A final direct effect of the war mentioned by some respondents was that wartime encourages the establishment of unstable marriages, in which violence is more likely to occur. This happens in two ways: firstly, a woman who loses her husband in the war may marry again quickly because she needs a man to support her, and is not always in a position to choose a non-violent partner; secondly, young girls are forced to marry fighters who, as we have seen above, may be particularly likely to use violence.

Some women lost their husbands during the war, and when they’ve lost their husbands they try to force themselves to other men because of their children. And when that happens and they have conflict, and the woman is normally hot-tempered because she’s stressed, and the man will say ‘Don’t come and bring your stress on me, I’m not the one that killed your husband’. (Freetown group 3)

### War-related transitions in women’s social roles

Across all of the four research locations, participants described how, during the war, women took responsibility for their families and became less dependent on men. This continued after the war, and women became more confident and more willing to challenge their partners.

One big change we are seeing is that women are engaged in a lot of income generating activities. Women are not idling now like before, initially women were dependent on their husbands, but now women are engaged in a lot of income generating activities, because a lot of them are breadwinners, and they pay school fees for their children. The other big change is that women’s voices, women are able to speak out. Before the war, women would not speak in such gatherings, it’s only men, but now we even speak as women in our own space and when we are with them we talk as well, so it’s a big change. Sometimes our voices are heard. We suggest, and our suggestions are being taken … Here, we have a woman as our local councillor. Before the war, that was not happening. Before the war you can’t even have a woman to aspire to paramount chieftancy, but that just happened. A woman contested to be paramount chief, and even though she didn’t win, she contested. Before the war, you can’t even dream about you, as a woman, being paramount chief here, let alone speak about it openly. Now women aspire as candidates for paramount chieftancy, and you see their pictures pasted on the walls. (Kailahun group 1)

In addition, women said that after the war, many national and international NGOs started working on issues of women’s rights and empowerment and established services for women affected by IPV. A great deal of sensitisation around these issues was conducted in communities, both for men and for women.

There was less agreement about men’s responses to this change and some diversity in the experiences reported. Some women said that these changes in women’s roles and behaviours had led to an increase in IPV, whilst others said that it had led to a decrease.

#### Transitioning social roles: decreases in IPV?

In some cases, women described that men began to recognize that the empowerment of women in terms of economic opportunities and participation in decision-making benefited not only women, but also men and the whole family. Some of these attitudes of men were attributed to engagement of men in the sensitisation conducted by NGOs. In these cases, women described how the new equality in relationships contributed to a decrease in IPV in the home.

Immediately after the war, domestic violence was high. But because of the sensitisation, the inflow of NGOs in Kailahun, most of them know what is what at this point, so it’s minimising. Even men are realising that if both the men and women contribute in the home, development will be faster than when he was alone providing. As a result, they are allowing women to engage in skills training activities. (Kailahun group 4)

Some respondents said that when women were able to contribute financially to the family, the pressure on men to provide for all their families’ needs was reduced, which in turn reduced the likelihood of IPV occurring.

I think that because women are going out and contributing, domestic violence is reducing. Most times, where the issue has been is when the man is 100% breadwinner. So if there is a day when he does not have, that’s where the beating comes. So now, if he brings half and I also bring half, he won’t beat me. (Kailahun group 2)

In addition, the fact that women have some financial independence was said to have a deterrent effect on men who know that their partners can leave if they are dissatisfied with the relationships. Women’s financial independence also was said to reduce IPV by giving young women the freedom to take their time in choosing the men they want to be with, rather than agreeing to be with the first man who offers to take care of them financially.

Education about women’s rights and the services available to women who are experiencing IPV were also said to deter men from beating their partners, because they knew that there were people their partners could call on for help, so there may be some consequences of their actions.

Domestic violence is reducing because of the empowerment, because prior to the empowerment, women were dependent on men in this area. So if a man said ‘get up! Go and clean my shoes’, we as women would be trembling because we felt that, if I say no, this man has my whole life. What if he says he isn’t going to feed me? What if he should leave me, what am I going to do? Right now I have something, I have the right to say, ‘yes papa, I’ll go and clean your shoe, but let me just do one or two things first’, because I have my own money. He will get vexed, because the first time he say ‘get up’ and we as women would be trembling to get up, but now he says ‘get up’ and you say ‘OK, I’ll do it later’, indeed he will get angry … But he won’t beat me because now I know my rights when he beat me, I will take him to the police. Before I didn’t know where to take him, but now I know where to take him. (Voinjama group 3)

The quotation above illustrates the interaction between the effects of empowerment and the importance of rights and services. If the woman above had financial independence, but did not know her rights and did not have access to services, she could be at increased risk of IPV. But she feels that the combination of financial empowerment, knowledge of rights, and access to services decreases her risk.

#### Transitioning social roles: increases in IPV?

Not everyone described social changes as positive. Rather, some women also reported that these same changes could sometimes lead to an increase in IPV. The women we spoke to said that some men saw women’s financial independence as a threat or a challenge and were therefore more likely to use IPV in an attempt to re-exert control over their partners.

Domestic violence has increased after the war, they won’t stop beating. As long as I’m finding my own money. Even if you beat me I will hide it [the money] … Even if you have your own money, they’ll say you are challenging them. It’s because you have your own money, that’s why you’re challenging them. (Kailahun group 3)

Some women said that men saw during the war that women were able to provide for their families, and after the war they expected women to continue to do this and became angry with women who did not. When men began to earn money after the war, some of our respondents said they did not use it to benefit the family since they expected women to provide for the family’s needs; they instead used it outside the family, for example to support girlfriends. This led to conflict within the family, as women challenged their partners about their failure to contribute and about their infidelity. Women’s education and empowerment increased the likelihood that women would indeed challenge their partners on these issues.

Prior to the war and right now, the men, they are very different. Before the war, whenever a man was working, you the woman in the home, he would give you food money, he would give you money for clothing, but right now, even if the man is working, he doesn’t give you money. Men these days, they are working, but because they have four or five girlfriends, they have the money but they spend outside the home. Whenever you try to talk about it, they beat you. Prior to the war, even if they had girlfriends, they were focused to support the home. Before the war, women were not doing business, they were not selling … Now the war has also opened the eyes of men, because when they go out they see other women doing businesses, so they expect you to also be doing it. (Monrovia group 3)

A number of women said that post-war, some men got jobs and began to earn money again. They observed that these men sometimes used IPV in order to exert control over their partners and re-establish themselves as breadwinners and heads-of-households.

Just before the war ended in 2003, men were submissive because they had nothing; they had no resources. Women were the ones who went out to bring food, only a very few brave men went out, the ones who went out were the women, to go in search of food. Now they feel like they’re now earning money, so they want to exert control over women. When they had nothing, they put themselves down, they had no control over women. (Monrovia group 3)

## Discussion

From the perspective of our respondents, women’s safety in the home in Sierra Leone and Liberia was influenced by cultural and financial factors, some of which shifted during wartime. These findings suggest that women’s financial dependence on their partners was a clear risk factor, not only triggering violence when women had to ask their partners for support, but creating a situation in which a man could assault his partner without any fear of her reporting him or leaving him. The accepted norm of men’s absolute authority in the home was said to lead to an accepted norm of IPV; the women we spoke to told us that men are socially permitted to respond to questioning or disobedience with violence. This fits with findings from other locations that frequently-cited reasons for marital violence relate to women behaving in a way unexpected of their gender, such as neglecting household chores, disobeying husbands and withholding sex from their partners [27,44].

While some women stated that men were equally violent before and after the war, many reported that the war seemed to make violence perpetrated by men even more accepted and prevalent. We heard that men who fought came to see violence as a normal response to frustrations; young boys grew up learning that violence was generally accepted, including with their partners; and that even men who did not fight experienced psychological distress that was often expressed in violence towards their partners. Although empirical research in this field is limited, studies have suggested a link between exposure to violence by armed groups and IPV [4,19,22]. In armed conflict, women may be exposed to both direct war experience and IPV [21]. Jewkes, Sen & Garcia-Moreno have also written that countries with a culture of violence, or where violent conflict is taking place, experience an increase in other forms of violence [15]. Conflict can impact levels of IPV through the disruption or destruction of protective structures for women, particularly kinship systems [25], as well as through the development of ‘pro-violence’ norms [7], an increase in economic problems that contribute to tensions within the family [25], and the psychological effects on men of their exposure to violent, distressing events.

A loss of social role and status is a common consequence of war and displacement, as people lose their jobs, access to land for farming, and their roles within their communities [28-30]. Status inconsistency theories claim that those who perceive their status within the family to be inconsistent with social norms may use violence as a strategy to compensate for loss of power.

Women are often able to maintain their traditional roles and responsibilities through conflict and displacement, and so maintain their gender identity, whilst men are more likely to lose their central roles, especially that of protector and provider, which makes it difficult to maintain their sense of themselves as men in the community and family [28,29]. These losses have been connected by some with a tendency towards marital conflict [26,27,33]. For example, Okello and Hovil attribute IPV in Northern Uganda to the men’s loss of traditional roles and identity due to displacement and an inability of men to care for their families [25]. People they interviewed said that men, unable to support their families, feel impotent, which leads them into a vicious cycle of anger and abuse. This is exacerbated by women becoming the main providers for their families.

According to our respondents, significant social change also occurred in Sierra Leone and Liberia as a result of the war, particularly in terms of changing roles of women. Women gained more financial independence and decision-making power, shifting longstanding cultural norms. Now that the war is over, communities are in transition as they adjust and react to the new social roles of women. It seemed that the change was having positive effects in some cases, with men accepting the changes and seeing the benefits. In other cases, the changes were threatening, leading to more IPV as men strove to regain control.

In general, the individual survivors we spoke to did not think that the war contributed to their own partner’s violence, but in group discussions women almost unanimously agreed that the war had had an impact on IPV. Reasons for this are unclear. A potential explanation is that group members could observe the general behaviour of the men around them and see changing patterns attributable to the war. However, this connection between IPV and an external factor, like war, may be less obvious in an individual. The extent to which the war contributed to violence varied greatly across relationships and interacted with individual-level characteristics. Perhaps women felt that attributing their partner’s behavior to the war was to excuse it in some way. As one woman said, ‘no, he’s just a wicked man’, when asked whether the war contributed to her partner’s violent behavior.

The ‘sanctions and sanctuary’ framework suggests that IPV may be highest where the status of women is in a state of transition: where women have a very low status, violence is not needed to enforce male authority, whilst where women have a high status, they will probably have achieved sufficient power collectively to change traditional gender roles [17]. According to this hypothesis, IPV is thus usually highest at the transition point. Abramsky et al. [45] suggest that the fact that we sometimes - but not invariably - observe increased IPV risk associated with the higher relative status of a woman (for example if she works and her partner does not) can be interpreted in the light of theories that risk of IPV may increase during periods of transition in gender relations. Women who step into new roles before background gender norms have shifted may be at increased risk of violence. Studies of the effects of micro-credit programmes aimed at empowering women have found mixed results in terms of the impact on IPV [45], with some finding that economic empowerment protects women from IPV whilst others report that women’s adoption of non-traditional roles creates tension in the home and precipitates IPV.

In some settings, gender roles do not change, even when circumstances no longer enable men to live up to their ideal role as providers or women to dedicate themselves exclusively to traditional household chores [46]. In Ghana, Amoakohene [47] writes that although many women now work full-time outside the home, traditional gender expectations mean that they also are required to carry out all the household tasks and to be subservient to their husbands. The women Amoakohene spoke to felt that this situation contributed to IPV, since they were over-stretched and unable to meet all the expectations that their husbands and families had of them. As stated above, failure to meet gender expectations is one of the most frequently given reasons for IPV.

The ways in which we found social changes around the roles of women in Sierra Leone and Liberia to interact with IPV were complex and not uniformly negative or positive, especially in the transitional, post-conflict period. Women in different circumstances were likely to experience the change in different ways, with the transitions leading to an increase in IPV for some women and to a decrease for others.

### Reflections on the research process

Some challenges were experienced during the data collection process, particularly regarding translation. The translators were all female IRC employees working with the Gender-Based Violence programme in the country and location in which we were conducting the research. This had advantages because they were familiar with the issues raised by the participants and the terminology used. However, they were not trained in translation, and this resulted in a tendency to summarise and use their own words rather than the participants’ words.

We must also acknowledge that all participants had some connection to IRC. Those who participated in individual interviews were receiving support from IRC. Whilst we recognise that this is a specific sub-group of women who experience IPV, for ethical reasons we choose to interview only survivors who already had access to support services. Those who participated in group discussions were not necessarily receiving direct services but were known personally or professionally by IRC staff. It may be that these women had participated in IRC awareness activities, so had more knowledge of IPV issues than other women in their communities. However, their opinions on the ways in which the conflict in their country may have affected IPV are not likely to be influenced by their connection with IRC since IRC awareness activities did not address this issue specifically.

As with all qualitative research, we cannot claim that the women who participated in this research are representative of the populations of interest, though they do reflect the diversity of those populations in terms of age, socio-economic status and ethnic group. It is worth noting, however, that since we spoke to relatively small numbers of women in each of the four locations, it is difficult to draw conclusions about whether differences between their perspectives were due to differences in the urban and rural contexts, differences between the Sierra Leone and Liberian contexts, or individual differences between our participants in the various locations. There were some indications of differences between the settings in terms of urban versus rural and country context, and future work with larger samples may help to draw these out.

An additional note on analysis is that in some studies, a second coder is used to check the reliability of the identified themes. However, in this case a single analyst coded the data, and reliability was checked by sharing the initial analysis with stakeholders in the field (members of the IRC team involved in the data collection; representatives of the participants; and other interested parties).

### Implications for practice

The transitions taking place in gender relations in post-conflict Sierra Leone and Liberia have been facilitated in part by the work of national and international organisations that have been active in the country since the end of the war (or earlier) and have been building awareness around women’s rights. The rate of change varies within communities and within families, and when women’s expectations change at a different rate to their partners’, this can contribute to tension and conflict in the home. It is particularly important at this time that women have services and support available. It is also crucial that organisations understand the dynamics of this tension and conflict in the particular context and base programmes on this understanding. To accelerate the rate of change in a community before this understanding is developed, or before services and support are in place, could be very damaging.

Our findings highlight both the potential negative and positive influence that war can have on IPV and women’s rights, particularly as expressed through decision-making and economic empowerment. Given this variation, programming should focus on both preventing and remediating the negative consequences *and* on promoting the positive social shifts; focusing solely on the negative may lead to a missed opportunity to increase the positive social change that is already occurring in some cases in the context of conflict.

Our results suggest that one main threat to women’s safety in terms of IPV is women’s financial dependence on their partners. When we asked participants what they thought would most help women who were experiencing IPV, or would help to prevent IPV, almost all said that activities to enhance the financial independence of women would have the most impact. As we saw earlier, however, enhancing women’s independence can be threatening to men and can increase IPV. It is essential, therefore, that economic empowerment and livelihoods programming, which seek to address women’s low status and perceived inferiority, take place alongside interventions to encourage men to recognise the value of women and the benefits of women’s participation in the economy and the home. The involvement of men in activities designed to prevent gender-based violence is important, and has been recognised by IRC and other organisations (see, for example, the work of Sonke Gender Justice [48]).

Economic interventions are also important for preventing IPV among young girls from poor families who are especially vulnerable to abuse because of their total dependence on the men they marry at a young age. Enabling girls in this situation to provide for themselves (or their families to provide for them) would be a good investment of resources.

It is also necessary for support services for women who experience IPV to be reliable and accessible, both so women have somewhere to go for help and to deter men who might previously have believed they could assault their partners and ‘no-one would ask any questions’.

## Conclusions

This study shows clearly that IPV is a significant problem for women in Sierra Leone and Liberia. Women perceive the causes of IPV to be linked with other difficulties faced by women in these settings, including their financial dependence on men, traditional gender expectations, and social changes that took place during and after the wars in those countries. The wars increased the use of violence by some men and boys who were directly involved in the conflict, as violence became for them a normal way of responding to frustrations and challenges. However, the war seemed to decrease the use of violence by other men who saw benefits of new roles of women and realized that women now had the independence to leave a violent relationship. The interactions between war experiences and financial and cultural issues are multi-faceted and not uniformly positive or negative. Programming by humanitarian organizations in post-conflict settings should consider both the need to protect women from the backlash of war, particularly related to their new financial independence and the opportunities to capitalize on some men’s positive reactions to women’s new roles. Future research will be important for developing and evaluating interventions to prevent and respond to increased violence and to promote the positive impacts that transition after war could have for women’s safety.

## Endnotes

^a^In this paper, we use the WHO definition of IPV: ‘behavior within an intimate relationship that causes physical, sexual, or psychological harm, including acts of physical aggression, sexual coercion, psychological abuse, and controlling behaviors’ (WHO, 2010: 11). This includes violence from a current or former partner.

^b^The IRC is an international non-governmental organization that implements programmes to prevent and respond to violence against women and girls in post-conflict settings.

## Abbreviations

IPV: Intimate partner violence; IRC: International rescue committee; WHO: World health organisation.

## Competing interests

Funding was provided by the NoVo Foundation to the International Rescue Committee. Two of the authors (Roesch; Lehmann) are employed currently by the IRC, and the second author (Puffer) was previously employed by the IRC. The first author (Horn) conducted this work as a paid consultant for the IRC. NoVo Foundation is not contributing to publication fees.

## Authors’ contributions

RH led the development of the methodology and interview guides, collected the data, drafted the manuscript, and led analysis. EP consulted on the initial design of the study and development of the methodology and data collection materials, and helped to draft the manuscript. ER and HL conceived of the project, consulted on the study design and methodology, and were involved in revising the manuscript. All authors read and approved the final manuscript.

## References

[B1] Republic of Liberia Truth and Reconciliation Commission Preliminary Findings and Recommendations, Vol. 1 2009 Monrovia http://trcofliberia.org/resources/reports/final/volume-one_layout-1.pdf

[B2] Sierra Leone Truth and Reconciliation Commission Sierra Leone TRC Reports 2004 Freetown http://www.sierraleonetrc.org/index.php/view-the-final-report/download-table-of-contents

[B3] Catani C Jacob N Schauer E Kohila M Neuner F Family violence, war and natural disasters: a study of the effect of extreme stress on children's mental health in Sri Lanka BMC Psychiatry 2008 8 33 18454851 10.1186/1471-244X-8-33 PMC2386780

[B4] Gupta J Acevedo-Garcia D Hemenway D Decker MR Raj A Silverman JG Pre-migration exposure to political violence and perpetration of intimate partner violence among immigrant men in Boston Am J Public Health 2009 99 462 469 18703450 10.2105/AJPH.2007.120634 PMC2661447

[B5] World Health Organisation Changing Cultural and Social Norms that Support Violence 2009 Geneva: World Health Organisation

[B6] Olayanju L Naguib RNG Nguyen QY Bali RK Vung ND Combating intimate partner violence in Africa: opportunities and challenges in five African countries Aggress Violence Behav 2013 18 101 112

[B7] Uthman O Lawoko S Moradi T Sex disparities in attitudes towards intimate partner violence against women in sub-Saharan Africa: Socio-ecological analysis BMC Public Health 2010 10 223 20429902 10.1186/1471-2458-10-223 PMC2873587

[B8] Kim J Motsei M “Women enjoy punishment”: Attitudes and experiences of gender-based violence among PHC nurses in rural South Africa Soc Sci Med 2002 54 1243 1254 11989960 10.1016/s0277-9536(01)00093-4

[B9] Ilika AL Women’s perception of partner violence in a Rural Igbo Community Afr J Reprod Health 2005 9 77 88 16623192

[B10] Kimuna SR Djamba YK Gender based violence: correlates of physical and sexual wife abuse in Kenya J Fam Violence 2008 23 333 342

[B11] Odero M Hatcher AM Bryant C Onono M Romito P Bukusi EA Turan JM Responses to and resources for intimate partner violence qualitative findings from women, men, and service providers in Rural Kenya J Interpers Violence 2014 29 783 805 24255067 10.1177/0886260513505706 PMC3910289

[B12] Speizer IS Intimate partner violence attitudes and experience among women and men in Uganda J Interpers Violence 2010 25 1224 1241 19758975 10.1177/0886260509340550 PMC2877756

[B13] UNFPA Gender Based Violence in Sierra Leone: A Case Study 2005 New York: UNFPA

[B14] Mann JR Takyi BK Autonomy, dependence or culture: examining the impact of resources and socio-cultural processes on attitudes towards intimate partner violence in Ghana, Africa J Fam Violence 2009 24 323 335

[B15] Jewkes R Sen P Garcia-Moreno C Sexual violence World Report on Violence and Health 2002 Geneva: World Health Organisation 149 181

[B16] Gelles RJ The violent home: a study of physical aggression between husbands and wives 1974 Beverley Hills: Sage

[B17] World Health Organisation/ London School of Hygiene and Tropical Medicine Preventing Intimate Partner and Sexual Violence Against Women: Taking Action and Generating Evidence 2010 Geneva: World Health Organisation/ London School of Hygiene and Tropical Medicine

[B18] Women’s Refugee Commission Peril or Protection: The Link Between Livelihoods and Gender-Based Violence in Displacement Settings 2009 New York: Women’s Refugee Commission

[B19] Catani C Schauer E Neuner F Beyond individual war trauma: domestic violence against children in Afghanistan and Sri Lanka J Marital Fam Ther 2008 34 165 176 18412824 10.1111/j.1752-0606.2008.00062.x

[B20] Clark CJ Everson-Rose SA Suglia SF Btoush R Alonso A Haj-Yahia MM Association between exposure to political violence and intimate-partner violence in the occupied Palestinian territory: a cross-sectional study Lancet 2010 375 310 316 20109958 10.1016/S0140-6736(09)61827-4

[B21] Usta J Farver JAM Zein L Women, war, and violence: surviving the experience J Womens Health 2008 17 793 804 10.1089/jwh.2007.0602 18537482

[B22] Hossain M Zimmerman C Kiss L Kone D Bakayoko-Topolska M KA DM Watts C Men’s and women’s experiences of violence and traumatic events in rural Côte d'Ivoire before, during and after a period of armed conflict BMJ Open 2014 4 e003644 10.1136/bmjopen-2013-003644 PMC3939650 24568959

[B23] Vinck P Pham P Association of exposure to intimate-partner physical violence and potentially traumatic war-related events with mental health in Liberia Soc Sci Med 2013 77 41 49 23219850 10.1016/j.socscimed.2012.10.026

[B24] Annan J Brier M The risk of return: Intimate partner violence in Northern Uganda’s armed conflict Soc Sci Med 2010 70 152 159 19853985 10.1016/j.socscimed.2009.09.027

[B25] Okello MC Hovil L Confronting the reality of gender-based violence in northern Uganda Int J Transitional Justice 2007 1 433 443

[B26] Carlson S Contesting and reinforcing patriarchy: an analysis of domestic violence in the Dzaleka Refugee Camp (Rep. No. 23) Working Paper 2005 Oxford: Queen Elizabeth House, Department of International Development, University of Oxford http://www.rsc.ox.ac.uk/files/publications/working-paper-series/wp23-contesting-reinforcing-patriarchy-2005.pdf

[B27] Horn R Exploring the impact of displacement and encampment on domestic violence in Kakuma refugee camp J Refugee Stud 2010 23 356 376

[B28] Payne L Food shortages and gender relations in Ikafe settlement, Uganda Gend Develop 1998 6 30 36 10.1080/741922625 12321534

[B29] Miller KE Rasco LM Miller KE, Rasco LM Preface The Mental Health of Refugees: Ecological approaches to healing and adaptation 2004 Mahwah, New Jersey: Lawrence Erlbaum

[B30] Wessells M Monteiro C Miller KE, Rasco LM Internally Displaced Angolans: a child-focused, community-based intervention The Mental Health of Refugees: Ecological approaches to healing and adaptation 2004 Mahwah, New Jersey: Lawrence Erlbaum 67 94

[B31] Schrijvers J Internal refugees in Sri Lanka: the interplay of ethnicity and gender Eur J Develop Res 1997 9 62 82

[B32] Walter JA Refugees and domestic violence: Model-building as a prelude to services research J Soc Work Res 2001 2 237 249

[B33] Ondeko R Purdin S Understanding the causes of gender-based violence Forced Migration Review 2004 19 30

[B34] CIA World Factbook Sierra Leone https://www.cia.gov/library/publications/the-world-factbook/geos/sl.html

[B35] Coker A Richter D Violence against women in Sierra Leone: Frequency and correlates of intimate partner violence and forced sexual intercourse Afr J Reprod Health 1998 2 61 72 10214430

[B36] Amnesty International Annual Report 2011: The State of the World’s Human Rights 2011 London: Amnesty International http://www.amnesty.org/en/region/sierra-leone/report-2011

[B37] UN data Liberia http://data.un.org/CountryProfile.aspx?crName=LIBERIA

[B38] Allen M Devitt C Intimate partner violence and belief systems in Liberia J Interpers Violence 2012 27 3514 3531 22610827 10.1177/0886260512445382

[B39] Sobkoviak RM Yount KM Halim N Domestic violence and child nutrition in Liberia Soc Sci Med 2012 74 103 111 22185910 10.1016/j.socscimed.2011.10.024

[B40] Johnson K Asher J Rosborough S Raja A Panjabi R Beadling C Lawry L Association of combatants’ status and sexual violence with health and mental health outcomes in postconflict Liberia JAMA 2008 300 676 690 18698066 10.1001/jama.300.6.676

[B41] International Rescue Committee Let Me Not Die Before My Time: Domestic Violence in West Africa 2012 New York: International Rescue Committee

[B42] Pavlish C Action responses of Congolese refugee women J Nurs Scholarsh 2005 37 10 17 15813581 10.1111/j.1547-5069.2005.00010.x

[B43] World Health Organisation Putting women first: Ethical and safety recommendations for research on domestic violence against women 2001 Geneva: World Health Organisation

[B44] Koenig MA Ahmed S Hossain MB Khorshed ABM Women’s status and domestic violence in rural Bangladesh: Individual-and community-level effects Demography 2003 40 269 288 12846132 10.1353/dem.2003.0014

[B45] Abramsky T Watts C Garcia-Moreno C Devries K Kiss L Ellsberg M Jansen H Heise L What factors are associated with recent intimate partner violence? Findings from the WHO multi-country study on women's health and domestic violence BMC Public Health 2011 11 1 17 21324186 10.1186/1471-2458-11-109 PMC3049145

[B46] Wyrod R Between women’s rights and men’s authority: masculinity and shifting discourses of Gender difference in Urban Uganda Gend Soc 2008 22 799 823 19862350 10.1177/0891243208325888 PMC2767117

[B47] Amoakohene MI Violence against women in Ghana: a look at women's perceptions and review of policy and social responses Soc Sci Med 2004 59 2373 2385 15450710 10.1016/j.socscimed.2004.04.001

[B48] Sonke Gender Justice http://www.genderjustice.org.za

